# Validation of macromolecular flexibility in solution by small-angle X-ray scattering (SAXS)

**DOI:** 10.1007/s00249-012-0820-x

**Published:** 2012-05-26

**Authors:** Michal Hammel

**Affiliations:** Lawrence Berkeley National Laboratory, Physical Biosciences Division, Berkeley, CA 94720 USA

**Keywords:** Small-angle X-ray scattering (SAXS), Macromolecular flexibility, Rigid-body modeling, Ensemble analysis

## Abstract

The dynamics of macromolecular conformations are critical to the action of cellular networks. Solution X-ray scattering studies, in combination with macromolecular X-ray crystallography (MX) and nuclear magnetic resonance (NMR), strive to determine complete and accurate states of macromolecules, providing novel insights describing allosteric mechanisms, supramolecular complexes, and dynamic molecular machines. This review addresses theoretical and practical concepts, concerns, and considerations for using these techniques in conjunction with computational methods to productively combine solution-scattering data with high-resolution structures. I discuss the principal means of direct identification of macromolecular flexibility from SAXS data followed by critical concerns about the methods used to calculate theoretical SAXS profiles from high-resolution structures. The SAXS profile is a direct interrogation of the thermodynamic ensemble and techniques such as, for example, minimal ensemble search (MES), enhance interpretation of SAXS experiments by describing the SAXS profiles as population-weighted thermodynamic ensembles. I discuss recent developments in computational techniques used for conformational sampling, and how these techniques provide a basis for assessing the level of the flexibility within a sample. Although these approaches sacrifice atomic detail, the knowledge gained from ensemble analysis is often appropriate for developing hypotheses and guiding biochemical experiments. Examples of the use of SAXS and combined approaches with X-ray crystallography, NMR, and computational methods to characterize dynamic assemblies are presented.

## Introduction

**Fig. 1:**
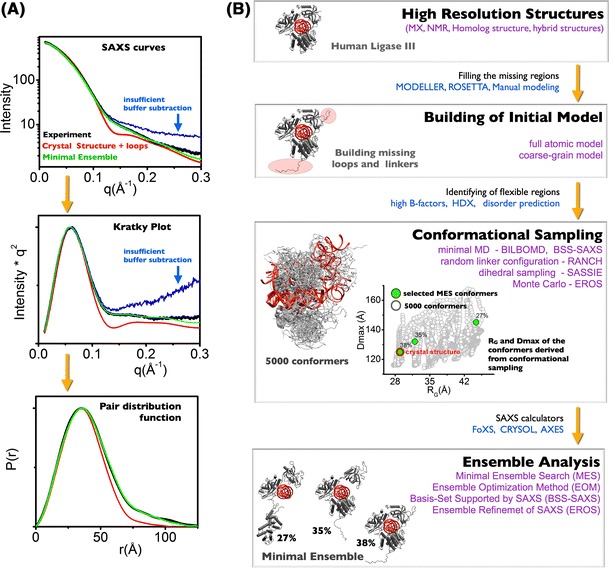
Validation of flexibility using SAXS curve (**a**) and rigid-body modeling (**b**). **a** Experimental SAXS profiles (*black* and *blue*) for the human DNA Ligase III (Cotner-Gohara et al. 2010) in a match with theoretical profiles calculated for the crystal structure (*red*) (Cotner-Gohara et al. 2010) and its dynamic model (*green*) obtained by BILBOMD and MES (Pelikan et al. 2009). The Kratky plot is used as the initial indicator of the flexibility. Baseline convergence necessary for assessing flexibility is misleading for the SAXS curve with insufficient buffer subtraction (*gray*). Pair distribution *P*(*r*) function calculated for the experimental (*black*) and the theoretical SAXS (*red*, *cyan*). Crystal structure, full-length and ensemble models used to calculate theoretical SAXS profiles are shown in the panel **a** (data adapted from Cotner-Gohara et al. 2010). **b** Schematic diagram of typical rigid-body modeling performing building of initial model, conformational sampling, and ensemble analysis

Current structure-based research has used high-resolution MX and NMR-derived structures to guide hypothesis-driven research. This has been effective for well-folded, compact enzymes, and has enabled atomic-level dissection of an enzyme’s active site. Nevertheless, estimates suggest that over 50 % of eukaryotic proteins contain significant functional unstructured regions (Vucetic et al. 2003) that are intractable to current structure-based model. Macromolecular flexibility is an important aspect of the regulatory mechanisms of biological systems (Henzler-Wildman and Kern 2007; Perry et al. 2010). MX, NMR, and electron microscopy (EM) are regarded as the most reliable methods for determination of structure; nonetheless, these techniques are limited by macromolecules with functional flexibility and intrinsic disorder (Fink 2005). Validation of macromolecular flexibility in solution by small-angle X-ray scattering (SAXS) has recently become a central tool in the new area of characterizing multi-state systems within structural biology (Bernado et al. 2007). Combining data from solution scattering with atomic resolution structures has the potential to address how specific complexes and flexibility drive biological processes (Putnam et al. 2007; Rambo and Tainer 2010a). Although SAXS has some inherent limitations, there is sufficient information within the one-dimensional scattering profile to distinguish between well-defined conformations and the conformational space occupied by a flexible assembly (Fig. 1). The theoretical basis for solution scattering has been the subject of an excellent review (Koch et al. 2003). Previously, I authored a review providing a general framework for experimental design, data processing, and data interpretation that combined SAXS with atomic-resolution structures from crystallography (Putnam et al. 2007). The purpose of this review is to discuss different tools and methods that have recently been developed for SAXS analysis of flexible multidomain assemblies.

## SAXS profile as a indicator of flexibility

**Fig. 2:**
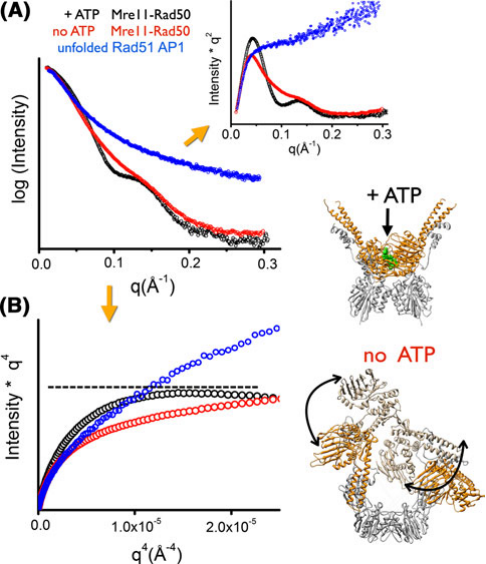
Detecting conformational flexibility. **a** SAXS data for the Mre11–Rad50 complex in both the presence (*black*), and absence (*red*) of ATP (Williams et al. 2011), and an exemplary intrinsically disordered domain Rad51 AP1 (*blue*). *Inset* Comparison of the Kratky plots for Mre11–Rad50 complexes does not confidently demonstrate flexibility of the complex in the absence of ATP (*black* and *red*). However, the Kratky plot of Rad51 AP1 (*blue*) is hyperbolic in shape, clearly demonstrating the full unfolded particle. **b** Porod–Debye plot illustrating changes in the Porod–Debye region. Loss of the plateau suggests Mre11–Rad50 becomes more flexible in the absence of ATP. Rigid and flexible states of Mre11–Rad50 are presented with crystal structure of Mre11–Rad50-ATPγS (Lim et al. 2011) and dynamic model of Mre11–Rad50 (Williams et al. 2011) (*left panel*). Data were adapted from Williams et al. (2011) and Rambo and Tainer (2011). Data for Rad51 AP1 were kindly provided by Gareth Williams at the Lawrence Berkeley National Laboratory

Recently Rambo et al. described the use of the Porod–Debye law as a powerful tool for distinguishing between rigid and flexible particles (Rambo and Tainer 2011). In particular, it was shown that for comparative SAXS experiments, application of the law can distinguish between discrete conformational changes and localized flexibility relevant to molecular recognition (Devarakonda et al. 2011; Williams et al. 2011). This approach aids insightful analysis of fully and partly flexible macromolecules that is more robust than traditional Kratky analysis (Porod 1982). Kratky analysis relies on visual inspection of the Kratky plot, which can be confounded by a limited observational *q* range (*q* < 0.2 Å^−1^), the presence of high experimental noise, or by non-ideal buffer subtraction (Fig. 1a). Intensity measurements at high scattering angles are exponentially more sensitive to the buffer – blank subtraction than measurements near the Guinier region. Therefore, small errors during the buffer – blank subtraction may confound the baseline convergence necessary for assessing flexibility by Kratky analysis (Fig. 1a). However, it has been shown that the Porod–Debye law resides within the low-resolution region of the SAXS profile, typically *q* < 0.15 Å^−1^, that is routinely well measured and not prone to buffer – blank subtraction issues. For example, Kratky analysis of the SAXS data collected for the ATP-free and bound forms of Mre11–Rad50 (Williams et al. 2011) did not clearly identify flexibility of the ATP-free state and rather led to the hypothesis that the particle is switching between two distinct conformational states, similar to PYR1(Nishimura et al. 2009). However, inspection of the Porod plot suggests a fundamentally different mechanism. In the presence of ATP, the complex forms a distinct particle with a sharp scattering contrast, as evidenced by the Porod plateau (Fig. 2b), and in the absence of ATP the particle becomes more flexible. In fact, inspection of the Porod–Debye region demonstrates a loss of the plateau, supporting the hypothesis that Mre11–Rad50 is flexible in the absence of ATP. These types of analysis provide qualitative information about conformational states that give credence to modeling the solution state as an ensemble of conformers.

## SAXS profiles provide more accurate atomic-level information about structures in solution without crystallographic constraints

Methods of analysis based on the concept of a single conformer cannot provide a complete three-dimensional model of dynamic proteins. Using a single “best” conformer to represent the ensemble at most provides a model representing an average of the conformations that exist in solution. Such a “best” single model of the macromolecular state can still be informative by helping guide a hypothesis regarding the macroscopic conformational state (Hammel et al. 2002; Iyer et al. 2008; Jain et al. 2009; Pascal et al. 2004; Williams et al. 2009). For example, if the crystal structure of a macromolecular assembly is known, a theoretical scattering profile can be calculated from the atomic coordinates. This provides the opportunity to evaluate several user-generated models (Fig. 1). If an extended conformer fits SAXS data better than a compact crystal structure, then an opening of the assembly in solution may be assumed (Nagar et al. 2006; Pascal et al. 2004; Yamagata and Tainer 2007).

Crystal packing forces are a selective pressure on a ensemble that typically promote a single conformer within the crystal lattice. Differences between crystal and solution states often reflect the presence of crystal packing forces (Cotner-Gohara et al. 2010; Datta et al. 2009; Duda et al. 2008; Nishimura et al. 2009; Stoddard et al. 2010) that can be used to gain new insights into a protein's flexibility (Nishimura et al. 2009). Direct comparisons of different conformational states with model SAXS profiles calculated from atomic-resolution structures have been quite successful in identifying and decomposing the relative fractions of conformers of a sample in solution, such as with the archaeal secretion ATPase GspE. The MX structure of the hexameric ring revealed a mixture of open and closed states of the individual subunits (Yamagata and Tainer 2007). In contrast, SAXS studies of GspE suggested a much different conformational state in solution. In the presence of the transition state ATP analogue, AMP-PNP, SAXS experiments suggest the enzyme’s subunits assume an all-closed state. In the next step of the catalytic cycle, the ADP-bound state, SAXS experiments suggest GspE exists as a mixture of all-closed and all-open states. The original crystal structure of alternating open–closed states in a ring failed to explain the SAXS experiments and raises significant questions regarding the proper biological state of the crystallized GspE. Crystal packing forces are structurally selective (Nishimura et al. 2009; Stoddard et al. 2010); consequently, a structural biology approach solely dependent on MX will be limited in scope.

## Accurate computation of SAXS profiles

**Fig. 3:**
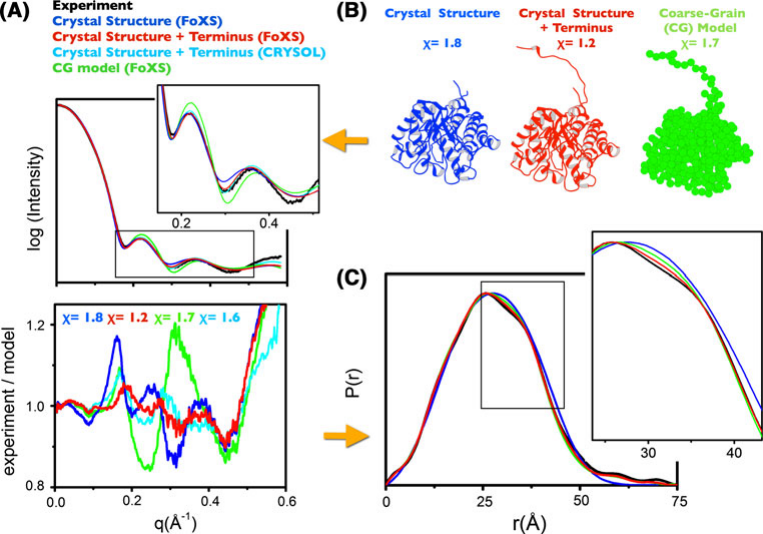
Accuracy of SAXS-profile calculations. **a** Comparison of the experimental scattering curves of cellulase Cel5A catalytic domain (*black*) with the theoretical curves for Cel5A crystal structure missing the C-terminal unfolded region (PDB 1EDG) (*blue*), full-atomistic model (*red*), and coarse-grain (CG) model (*green*), shown in panel **b**. *Bottom panel* The discrepancy between theoretical and experimental profiles is calculated as Intensity_(experiment)_/Intensity_(model)_. Please note the large discrepancy for the CG model (*χ* = 1.7) and crystal structure (*χ* = 1.8) in comparison with the full-atomistic model calculated by FoXS (*χ* = 1.2). Better profile matches are obtained by calculating explicit atom distances (FoXS *χ* = 1.2) in comparison with the SAXS profile calculated by spherical harmonics using CRYSOL—Linux version 2.7 (*χ* = 1.6). **c** *P*(*r*) functions calculated for SAXS profiles shown in **a** have been calculated by use of the software GNOM (Svergun 1992). The production and purification of the cellulase Cel5A catalytic domain has been described elsewhere (Fierobe et al. 2002). SAXS experiments were performed at the European Synchrotron Radiation Facility (Grenoble, France) on beamline ID02 as described by (Hammel et al. 2004a)

High-quality SAXS experiments from advanced instrumentation (Hura et al. 2009) lead to more precise data and confident assignment of the conformational state(s) of a given sample. Notwithstanding instrumentation developments, accurate calculation of a SAXS profile is essential for the accuracy of solution structure modeling. Several methods are available to calculate SAXS profiles from atomic models, and differ in the use of the inter-atomic distances, estimation of excluded volume, treatment of the hydration layer, or background adjustment (Grishaev et al. 2010). Calculation of an SAXS profile from atomic coordinates requires spherical averaging that can be efficiently accomplished by representing a macromolecule in terms of inter-atomic distances (Schneidman-Duhovny et al. 2010; Zuo et al. 2006) or by using spherical harmonic reconstructions (Grishaev et al. 2010; Liu et al. 2012; Svergun et al. 1995). Explicit calculation of inter-atomic distances with solX software (Zuo et al. 2006) requires more intensive computation, but results in good agreement throughout the large *q* range with experimental scattering profiles (Putnam et al. 2007). Calculating profiles for anisometric shapes or unfolded regions is also more problematic for spherical harmonic reconstructions (reviewed by Putnam et al. 2007) and inaccuracies in fitting can be compensated by over-adjustment of excluded volume or the density of the hydration layer. As the data quality becomes extraordinary good, full atomistic models are required for accurate interpretation of the experimental SAXS profiles (Fig. 3). In this example of a high-resolution experimental SAXS of the cellulase Cel5A catalytic domain, explicit calculations using inter-atomic distances of several models demonstrate that the calculation of accurate profiles may detect small unfolded regions (Fig. 3). SAXS can detect these unstructured regions only because they affect the overall/globular shape of the protein. However, the example presented clearly shows the kind of information content stored in the SAXS profiles or its *P*(*r*) functions derived from them (Fig. 3b). The fact that the full-atomistic model is important to match experimental data has been further shown by analysis of 19 proteins containing a 19-residue His tag (Hura et al. 2009). His tags increase *D*_max_, and should be modeled explicitly with available core atomic models.

Fitting theoretical models to SAXS profiles requires that a measure be established for determining the agreement between two scattering curves. I am not convinced that a “best” measure of assessing agreement between experimental and theoretical curves has been adequately developed. The standard *χ* clearly weighs the lowest resolution data most strongly. The *χ* values become less informative as the high resolution SAXS profiles with “low-noise” are used to fit atomistic models. For additional assessment of the quality of model-data agreements I suggest displaying the discrepancy by using the ratio calculated as *I*_experiment_/*I*_model_. This residual-ratio clearly displays discrepancies in the important small *q* region whereas the standard log_10_-based presentation of log (*I*) versus *q* frequently does not (Figs. 3a and 5).

**Fig. 4:**
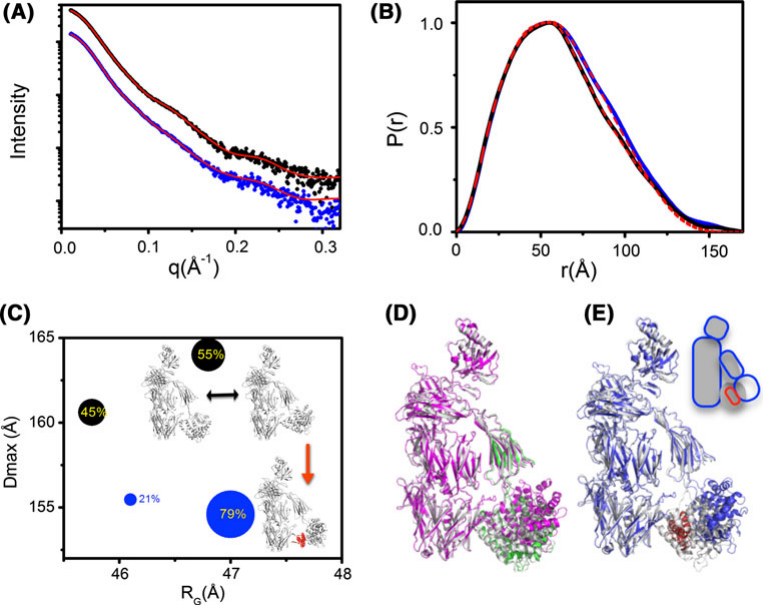
Efb-induced conformational changes in human complement C3b as revealed by SAXS. **a** Experimental scattering curves for free C3b (*black*) and in the complex with extracellular fibrinogen-binding protein (Efb) from *Staphylococcus aureus* (C3b/Efb) (*blue*) were fit to MES model (*red line*). **b** *P*(*r*) functions indicate conformational changes between C3b (*black*) and C3b/Efb (*blue*), where broadening of *P*(*r*) for C3b/Efb-C is consistent with reorientation of the CUB-TED domain. *P*(*r*) from the atomic MES models is shown as a *red dashed line*. **c** Comparison of *R*_G_ for the two predominant MES conformers of either C3b (*black*) or C3b/Efb (*blue*) as obtained by BILBOMD sampling with their maximum dimensions (*D*_max_). Dot sizes represent the fraction ratio of the two conformers in each group. Rigid-body modeling-derived C3b conformers are shown in *gray* with Efb highlighted in *red*. (**d**, **e**) Superposition of the BILBOMD-MES-derived conformers of free C3b (**d**, *magenta* and *green*) and C3b/Efb (**e**, *blue*/*red*) with the crystal structure of C3b (*gray*). The *inset* shows a schematic representation of the proposed domain rearrangements. Data were adapted from Chen et al. (2010)

Better quality experimental data promotes the need for increased accuracy and computations of SAXS profiles. By using explicit-all atom distances (Schneidman-Duhovny et al. 2010) and water models to account for the effect of solvent (Grishaev et al. 2010) superior fits between experimental high resolution structures and SAXS data are obtained (Fig. 3a). The explicit representation of the molecule is particularly useful for multidomain-flexible assemblies, which frequently adopt highly anisometric shapes (Grishaev et al. 2010). The FoXS algorithm explicitly computes all inter-atomic distances that include the first solvation layer based on the atomic solvent accessible areas (Fig. 3). As FoXS is available through a web server, it enables uploading and simultaneous analysis of a collection of atomic coordinate input files against experimental data. In combination with the MES (Pelikan et al. 2009) that is also part of the suite, the user is provided with powerful tools to identify the heterogeneity or flexibility of the experimental system. These powerful analytical techniques, together with advanced instrumentation, have been the basis for visualizing minimum conformational changes in human complement C3b (Chen et al. 2010) (Fig. 4).

## Modeling of the conformational space

Although comparison of model SAXS profiles with the experimental data is one of the most straightforward applications of SAXS, the uniqueness of arrangements of atomic resolution structures that fit SAXS data must also be evaluated. The determination of multidomain or subunit assemblies using rigid-body modeling in conjunction with SAXS data involves preparing a large number of possible atomic models and comparing them with experimental data. The models can either be refined directly against experimental data (Petoukhov and Svergun 2005) or prepared independently using the SAXS data as a filter to select the “best fit” model(s) (Boehm et al. 1999; Forster et al. 2008). The biggest challenge in trying to model flexible multidomain systems using SAXS data is to avoid over-fitting. Most commonly, over-fitting can be detected by visually inspecting the selected models and examining for large unfolded regions or unrealistic inter-domain distances. Extremely elongated or partially unfolded structures may contribute to inappropriate “successful fits” of experimental data derived from aggregated or heterogeneous samples (reviewed by Putnam et al. 2007). For example, studies of mammalian lipoxygenase illustrate the need for establishing monodispersity of sample in cases where domain flexibility is proposed (Dainese et al. 2005; Hammel et al. 2004b; Shang et al. 2011). In early studies the discrepancy between the experimental curve of mammalian lipoxygenase and the profile calculated from the atomic coordinates were interpreted in terms of a very large movement of the N-terminal domain (Hammel et al. 2004b). In a recent study, however, Shang et al. (2011) found that mammalian lipoxygenase, besides its flexible N-terminal domain, forms a transient dimer that also leads to an elongated SAXS signal. Therefore, samples that are suspected of possessing intrinsic flexibility must be carefully characterized to ensure monodispersity before SAXS modeling (Rambo and Tainer 2010b).

**Fig. 5:**
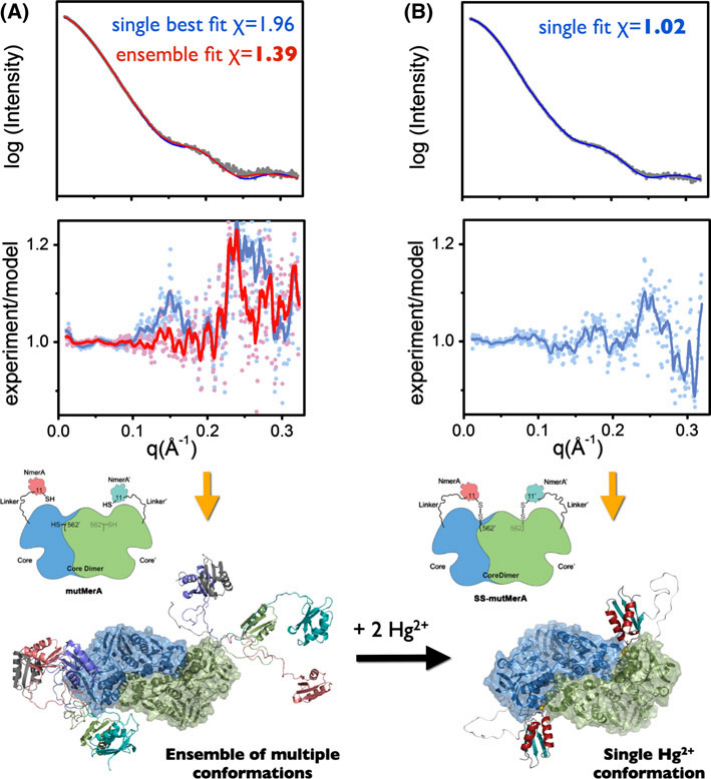
Solution structure modeling of intramolecular Hg^2+^ transfer between flexibly linked domains of mercuric ion reductase (MerA). **a** Comparison of experimental and calculated scattering profiles for full-length MerA (mutMerA). Experimental SAXS data (*gray*), single best-fit conformation to the experimental scattering profile with *χ* = 1.96 (*blue line*), and combined profile from five contributing conformations identified by MES (*red line*) with *χ* = 1.39. Residuals calculated as *I*_experiment_/*I*_model_ are shown at the *bottom*. Superposition of the five models identified by MES with the metallochaperone-like N-terminal domains in a different color weighted by the factors 0.40 (*pink*), 0.29 (*green*), 0.16 (*cyan*), 0.08 (*purple*), and 0.07 (*gray*). **b** Experimental SAXS data for the disulfide-cross-linked handoff complex (SS–mutMerA) (*gray*) and calculated scattering data for the single best-fit conformation *χ* = 1.02 (*blue line*). Residuals *I*_experiment_/*I*_model_ are shown as *blue dots* and as a *blue line* for smooth residuals. *Inset* shows the schematic representation of mutMerA and S–S-mutMerA. Data were adapted from Johs et al. (2011)

A number of techniques have been used to generate realistic atomic models that sample conformational space of multi-modular proteins. Monte Carlo simulation (Forster et al. 2008; Rozycki et al. 2011) based on exploration of the dihedral angles in connection regions (Akiyama et al. 2004; Curtis et al. 2012), torsion/Cartesian simulated annealing (Schwieters et al. 2010), and minimal molecular dynamics (minimal MD) (Boehm et al. 1999; Hammel et al. 2005; Yang et al. 2010) may all be used. In the early years of rigid-body modeling the Perkins group developed constrain molecular modeling. This approach was applied to solution structure determinations of human and chimeric antibodies (reviewed by Perkins and Bonner 2008). The technique uses a large number of conformers that are built with directed MD computations applied only to the inter-domain connections. These models are filtered on the basis of their agreement with properties extracted from experimental SAXS curves, for example the radius of gyration, radius of gyration of cross sections, and the overall fit of the theoretical scattering from the model to the experimental data (Abe et al. 2010; Aslam and Perkins 2001; Gilbert et al. 2006; Khan et al. 2010; Li et al. 2010). Constrained modeling confirms the experimental data analysis and produces families of best-fit models. Although these molecules are most likely an ensemble with a wide range of conformations, the selected best fit conformers are sufficient to reveal conformational switching or flexibility. The recently developed BILBOMD approach uses a similar minimal MD strategy and describes the final model as a population-weighted ensemble selected from the entire pool of conformers (Pelikan et al. 2009) (Figs. 4 and 5).

Conformational sampling may also be performed with simplified coarse-grain (CG) models, where amino-acid residues are presented as spherical beads centered at corresponding Cα atom positions (Rozycki et al. 2011; Yang et al. 2010). Although extremely simplified, CG incorporates the main generic features and folding data of the protein under investigation. The CG models are used to not only speed up the production phase of conformational sampling but also to speed up the SAXS calculation. However, CG models are coarse representations, and it has been shown that full atomistic models are required for accurate calculation of SAXS profiles (Grishaev et al. 2010) (Fig. 3). Particularly for modeling flexible assemblies, the atomistic representation is essential for accurate representation in solution when the particles deviate from a canonical globular shape (Fig. 3).

## Distance constraints in rigid-body modeling

Accurate assignment of the flexible regions is crucial to realistic conformational sampling. In most cases, analysis of high-resolution structures can indicate plausible regions of structural flexibility (Chen et al. 2010). Missing electron density (Bernstein et al. 2009; Biersmith et al. 2011; Hammel et al. 2007a; Hammel et al. 2010b) or regions with a high isotropic atomic displacement factor (ADF also called the B-factor) (Duda et al. 2008; Williams et al. 2011) are useful indicators of flexible regions. Empirical determination of flexible regions can be achieved by hydrogen–deuterium exchange mass spectrometry (HDX) which specifically follows changes in conformational states of proteins. For example, HDX clearly assigned the flexible region in the complement C3b molecule after its activation (Hammel et al. 2007b). This HDX experiment guided SAXS based rigid-body modeling used to visualize the C3b molecule as a highly dynamic system. SAXS modeling also revealed that C3b flexibility may be effected by an allosteric inhibitor, for example the extracellular fibrinogen-binding protein (Efb) from *Staphylococcus aureus*. This is the first reported evidence that the system is controlled by allosteric inhibitors and supports new views in which modulators may stabilize preexisting intrinsic conformations rather than inducing completely new domain arrangements (Chen et al. 2010) (Fig. 4).

Furthermore, realistic models may by derived by incorporating additional information about the system in question, for example known distance constraints. Techniques that provide local distance and angle information, for example Förster resonance energy transfer (FRET) (Rochel et al. 2011) and NMR (Bertini et al. 2008; Mareuil et al. 2007) may provide useful restriction in inter-domain movement and guide conformational sampling. The rigid body/torsion/Cartesian simulated annealing strategy developed by Grishaev et al. (Grishaev et al. 2005; Mittag et al. 2010) integrated both NMR and SAXS observations into a unique synergistic method for atomistic modeling. From NMR, residual dipolar coupling (RDC) data were used to orient the symmetrically related protein domains relative to the symmetry axis of the protein core whereas translational, shape, and size information was provided by SAXS (Schwieters et al. 2010). FRET in combination with SAXS guided rigid-body modeling to aid elucidation of the structural basis of the role of DNA in the spatial organization of nuclear hormone receptors in complex co-activators (Rochel et al. 2011). Distance restraints may also be generated from simple biochemical techniques, for example site-direct mutagenesis. For example, integrated site-directed mutagenesis and SAXS combined with conformational sampling of DNA binding sites were used to determine the DNA-binding properties of mPNK (Bernstein et al. 2009) and reveal the intramolecular metal ion transfer between flexibly-linked domains of mercury ion reductase (Johs et al. 2011) (Fig. 5).

## The conformational ensemble

Although exhaustive conformational sampling significantly increases the number of realistic models to be used for modeling experimental SAXS data, a single best-fit conformation may be incapable of explaining the observed SAXS profile. The lack of convergence of a single best-fit conformation has been shown to correlate with conformational disorder rather than a limitation of the search space algorithm (Pelikan et al. 2009). In the case of scattering from a heterogeneous population, the measured scattering is derived from the population-weighted thermodynamic ensemble, and the interpretation of dynamic systems requires analysis beyond “best fit” conformations (Figs. 4 and 5). In recent years, new SAXS modeling techniques have been developed to describe dynamic systems in terms of ensembles of structures (Bernado et al. 2007; Pelikan et al. 2009; Rozycki et al. 2011; Yang et al. 2010). Four promising approaches for modeling the ensemble are pushing SAXS into an exciting new direction, the ensemble optimization method (EOM) (Bernado et al. 2007), minimal ensemble search (MES) (Pelikan et al. 2009), ensemble refinement of SAXS (EROS) (Rozycki et al. 2011), and basis-set supported by SAXS (BSS-SAXS) (Yang et al. 2010). Because of the nearly infinite number of conformations that can be adopted by flexible proteins in silico, obtaining meaningful models requires the development of robust statistical approaches that determine the probability a particular multi-conformational equilibrium will exist (Bertini et al. 2010). Again, a common problem with multi-conformational analysis is over-fitting, which occurs when an ensemble model describes noise or aggregation in the experimental system, rather than the desired underlying relationship. MES avoids over-fitting by asserting the minimum number of states that could be distinguished from SAXS data. In addition, to avoid over-fitting the data with the multiple conformations (Bernado et al. 2007), a quantitative description of the ensemble also requires the weighting of each conformer's distribution (Pelikan et al. 2009; Yang et al. 2010). For the purpose of avoiding over-fitting of raw data, Rozycki et al. constructed a pseudo free energy scheme to refine the statistical weights attributed to configurations generated by simulation (Rozycki et al. 2011). These SAXS ensemble methods seem enormously successful on the basis of analysis of several key biological systems: identification of the correct subunit positions for full-length Ku (Hammel et al. 2010b), demonstration of the flexibility in full-length polynucleotide kinase (Bernstein et al. 2009), establishment of the configurational space of Lys-63 linked tetraubiquitin (Datta et al. 2009), elucidation of the flexibility mode in a Ubiquitin-PCNA complex involved in DNA replication and repair (Tsutakawa et al. 2011), and describing the partially unfolded state of XRCC4 (Hammel et al. 2010a) and XRCC4-likes proteins (Hammel et al. 2011).

## Conclusions and prospects

Structural biology now recognizes that partially populated states are crucial to biological function. The single conformation description of a macromolecule is only a snapshot of a macromolecular ensemble. We have seen that integrative methods that utilize NMR and MX with SAXS are proving to be essential for providing a larger description of the macromolecular ensemble. Using SAXS data as a source of experimental restraints for modeling macromolecular flexibility is an exciting and relatively underdeveloped discipline. SAXS data can provide important experimental feedback, and can be extended to include dynamic conformational changes characterized by time-resolved experiments. Time-resolved measurements require very high X-ray flux and fast detectors designed for rapid electronic shuttering. Both are now available, and SAXS, unlike traditional NMR and fluorescence experiments, is not affected by molecular rotation times, so time-resolved SAXS can be performed in an equivalent manner to the traditional static experiments. The development of the approaches for characterizing highly fluctuating conformational equilibria on the basis of traditional static experiments are becoming essential in the description of intrinsic dynamic biomolecular systems (Bernado and Blackledge 2010). Macromolecular machines with flexible and unstructured regions are now tractable to direct structural investigation (Bernado 2010; Bernado and Svergun 2012). These are some of the reasons why SAXS-based solution structure modeling of flexible macromolecular assemblies are gaining popularity and will be used in the future to elucidate the roles of dynamic equilibrium in biological processes (Rambo and Tainer 2011). A natural complement to the global shape and conformation from SAXS will be residue-level information from advancing techniques of enhanced hydrogen–deuterium exchange mass spectrometry, which can approach single-residue resolution as shown for the photocycle changes of photoactive yellow proteins (Brudler et al. 2006). Thus, SAXS is well positioned to become an important technique, with new weak-field aligned NMR and fluorescence experiments that can probe samples in the biologically interesting millisecond time frame. With appropriate resources for directed efforts, SAXS can provide complementary experimental data on flexibility in macromolecular interactions with widespread effects.
